# PMMA Bone Cement: Antibiotic Elution and Mechanical Properties in the Context of Clinical Use

**DOI:** 10.3390/biomedicines10081830

**Published:** 2022-07-29

**Authors:** Sebastian Philipp von Hertzberg-Boelch, Martin Luedemann, Maximilian Rudert, Andre F. Steinert

**Affiliations:** 1 Department of Orthopaedic Surgery, University of Wuerzburg, Koenig-Ludwig-Haus, 11 Brettreichstrasse, 97074 Würzburg, Germany; m-luedemann.klh@uni-wuerzburg.de (M.L.); m-rudert.klh@uni-wuerzburg.de (M.R.); andre.steinert@campus-nes.de (A.F.S.); 2 Rhön Klinikum, Campus Bad Neustadt, EndoRhoen Center for Joint Replacement, Teaching Hospital of the Phillipps University Marburg, Von Guttenberg Str. 11, 97616 Bad Neustadt, Germany

**Keywords:** spacer, bone cement, PMMA, polymethylmethacrylate, periprosthetic infection, antibiotic elution

## Abstract

This literature review discusses the use of antibiotic loaded polymethylmethacrylate bone cements in arthroplasty. The clinically relevant differences that have to be considered when antibiotic loaded bone cements (ALBC) are used either for long-term implant fixation or as spacers for the treatment of periprosthetic joint infections are outlined. In this context, in vitro findings for antibiotic elution and material properties are summarized and transferred to clinical use.

## 1. Introduction

**Figure 1:**
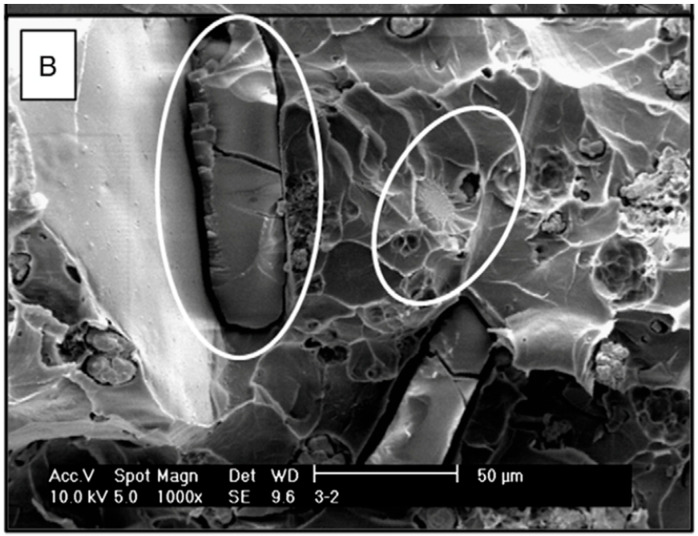
SEM micrograph of the fracture surface of a PMMA with vancomycin (1000×). The circles indicate the crystals. Taken from Paz et al. [4]; reprinted with permission from Elsevier.

Antibiotics and bone cements are ubiquitously used in orthopedic and trauma surgery. Methylmethacrylate (MMA) is the main component of bone cements [1,2]. MMA molecules bind to long chains by radical polymerization to polymethylmethacrylate (PMMA). Further components are radiopacifiers, coloring agents, antibiotics, and additives that modulate the polymerization process. The compositions vary depending on the cement type and brand. For clinical use, bone cements are prepared as two-component systems for easy application. After mixing the two components, i.e., the cement powder and the liquid, the cement changes from a doughy to a solid state. During this curing process, PMMA bone cements exhibit almost unlimited plasticity. After this curing process, the solid cement has mechanical properties, which makes it suitable for implant fixation, but also for the filling of anatomic voids or to provide buttress functions [3]. Additionally, PMMA bone cements can be used as a drug carrier for added antibiotics (Figure 1) [1].

To a certain extent antibiotics added to the starting components before mixing will be released from the cured cement. Thus, bone cements combine two basic principles for surgery of the musculoskeletal system: stable fixation and anti-sepsis. Because of these qualities, antibiotic loaded bone cements (ALBC) have gained outstanding significance in the field of arthroplasty. In this context, ALBCs are mainly used for two indications, i.e., infection prophylaxis and treatment of implant associated infections [5].

This literature review summarizes the use of ALBCs in arthroplasty. Their application for implant fixation and as spacers is outlined. The in vitro results on antibiotic elution and mechanical properties of ALBCs are discussed from a clinical point of view.

## 2. Indications for ALBCs in Arthroplasty

Two different indications for ALBCs need to be discriminated [6].

ALBCS are predominantly used for long-term implant fixation. In the 1970s, Wahlig and Buchholz from Hamburg performed the first antibiotic elution tests from PMMA [1,7] and then implemented ALBCs for implant fixation in the following years [8]. To date, cemented implant fixation is routine in total hip arthroplasty (THA) and total knee arthroplasty (TKA) [9,10]. It is evident that fixation with ALBCs reduces the risk of implant infection compared to cemented fixation without antibiotics [11]. However, the routine use of ALBCs for primary TKA and THA is still debated because of concerns about resistance formation and aseptic loosening [12,13]. Nonetheless, in several countries ALBCs are used by default for cementing primary TKAs and THAs [14,15]. In this context, ALBCs are used to provide lasting implant fixation with antibiotic prophylaxis.

The second indication for ALBCs is the two-stage exchange of an infected prosthesis. Herein, ALBCs are used in the form of temporary spacers. During the first surgery, the infected prosthesis is removed and replaced with the spacer. When the infection has resolved the spacer is removed and the joint is reconstructed with a new prosthesis in a second operation. In this context, ALBCs fill voids, provide temporary stability, and serve as a drug carrier for local antibiotic administration. The ALBC- spacer yields high local antibiotic concentrations that cannot be achieved by systemic administration, and at the same time minimizes the risk of toxic side effects [5,6,16,17]. German registry data indicates that about 10% of all revisions are performed as two-stage exchanges [10]. Although the two-stage exchange has variable success rates ranging between 50 and 100% [18], it is regarded as the gold standard treatment for periprosthetic joint infection [14].

## 3. Mechanical Properties of Hardened PMMA Cement

### 3.1. Requirements

PMMA cements have to provide sufficient strength against bending, impact, tension, torsion, and shear forces [11]. The International Organization for Standardization (ISO, Geneva, Switzerland) defines compressive strength, bending strength, and bending modulus as the key indicators for mechanical properties of polymerized cements [19]. The bending modulus measures the bending extent and the bending strength measures the load until failure at two defined load forces [19]. Commercially available ALBCs must provide a bending modulus of at least 1800 MPa and a bending strength of 50 MPa [11]. The compression test evaluates the maximum compressive force before the plastic deformation of cylindric specimens occurs [19]. The mechanical properties of commercially available bone cements differ depending on their brand and composition. Compressive strengths range commonly between 80 and 100 MPa. However, all commercially available ALBCs must meet the ISO requirement to withstand a compression force of at least 70 MPa [11].

Various in vitro studies investigated the effects of antibiotic loading on mechanical properties. However, comparability is often limited because they vary considerably in their set up. There are only a few studies that directly compared the effect of manual antibiotic loading while taking into account the cement brand or type. In a study by the authors, compression strengths of Palacos^®^ R+G (Haereus, Hanau, Germany) and Copal^®^ spacem (Haereus, Hanau, Germany) were compared after antibiotic loading. Although slight differences were observed, these were not significant [3]. To date, it is not determined which bone cement is most suitable for additional antibiotic loading.

### 3.2. Type of Antibiotic

Antibiotic loading reduces mechanical stability because antibiotic molecules interfere with the curing of the cement. Particularly liquid antibiotics interfere extensively with the polymerization process. For example, Hsieh et al. reported a reduction of compressive strength of 37% when liquid gentamicin was added to Simplex bone cement (Stryker, Kalamazoo, MI, USA) [20]. Thus, as a principle, only crystalline antibiotics should be used and added to the powder component [6,16]. The effect on mechanical properties is not only depended on the physical state of the antibiotic, but also on its type. Some antibiotics interfere more with the polymerization process than others. For example, rifampicin, although in a crystalline state, can inhibit the complete curing of PMMA bone cements [21]. Therefore, only antibiotics should be used that were found in vitro to be appropriate for manual loading. A broad spectrum of crystalline antibiotics was denominated for the loading of bone cements during the International Consensus Meeting in 2018 (Table A1 in the Appendix A) [14].

### 3.3. Proportion of Antibiotic in the Bone Cement

The more antibiotic is added, the more the stability of the cured cement will be reduced. However, the maximum proportion of antibiotic in the cement powder is not conclusively established. Although many studies investigated the effect on stability with variable proportions of antibiotic in the bone cement [21,22,23], only a few studies were conducted in accordance with the ISO 5833 for the testing of acrylic bone cements. In our own study with Palacos^®^ R+G and Copal^®^ spacem, an antibiotic proportion of 2.5% in the cement powder reduced compression strength to a value close to the required 70 MPa [3]. Lilikakis et al. investigated the effect of vancomycin addition to the bone cements Palamed^®^ (Haereus, Hanau, Germany) and Copal^®^ G+C (Haereus, Hanau, Germany). After an addition of 5% vancomycin, the compression strength remained significantly above 70 MPa for both cements. Even with an addition of 10% vancomycin, compressive strength was reduced only by 18.15% and 17.48%, respectively, and the compressive strengths of both cements remained above the 70 MPa cut-off value. However, only the Palamed^®^ cement type was significantly above this limit [22]. Therefore, the changes to mechanical properties induced by antibiotic loading are inter alia dependent on cement brand, cement type, and proportion of antibiotic. Currently, manual antibiotic loading up to a cumulative antibiotic proportion of 5–10% in the cement powder is considered appropriate for the preparation of a temporary spacer [6,16,22,24]. This assessment is based on the ISO standard requirements. However, these requirements apply for permanent implant fixation. Because the mechanical needs after spacer implantation can be managed by postoperative restricted weight bearing, other authors approve of a cumulative antibiotic proportion of up to 20% for spacers [1].

### 3.4. Loading Technique

Two different and in part contradictory recommendations for adding the antibiotic to the cement powder are discussed: Kuhn et al. propagate a thorough homogenization of the antibiotic powder and the cement powder. The crystalline antibiotic is ground in a mortar and then added to the cement powder in several steps. This mixing technique is supposed to provide consistent and reproducible cement properties after hardening [6]. In contrast, Parvizi describes adding the antibiotic to the cement powder all at once resulting in only rough homogenization. This technique is thought to provide maximum antibiotic elution after hardening due to the formation of antibiotic clumps in the cement [25]. Laine et al. compared the effects of different loading techniques that give varying levels of homogenization. Dispensing with the homogenization process almost entirely induced pore formation. Despite the pore formation, subsequent tests of mechanical strength did not reveal a significant difference [26]. Theoretically, the different mixing strategies should lead to different mechanical properties of the manually loaded PMMA cement. However, the clinical significance of the loading technique remains to be confirmed.

### 3.5. Legal Implications

The legal implications of manually loading bone cements intended for TKA or THA fixation have to be considered. Aseptic loosening is still the number one reason for implant failure after TKA and THA [9,10]. Antibiotic elution reduces mechanical strength of ALBCs over time [11]. In our aforementioned study on the cements Palacos^®^ R+G and Copal^®^ spacem, antibiotic loading with more than 10% of vancomycin led to significant compressive strength reduction after 4 weeks of antibiotic elution [3]. Comparable observations are described by Lee et al. for other cement brands. After 336 h of aging, significant reduction was noted for Simplex^®^ P, Palacos^®^ and CMW^TM^ (Depuy, Warsaw, IN, USA) loaded with 10% of vancomycin [27]. Thus, antibiotic elution reduces mechanical strength. With regard to manual loading, the extent of this effect cannot be predicted. By manually loading, the surgeon becomes the responsible manufacturer of the bone cement because he alters its mechanical properties. Therefore, the manual loading of bone cements intended for long-term prosthesis fixation cannot be recommended. Instead, the surgeon should use commercially available ALBC (Table A2 in the Appendix A) with consistent mechanical properties in combination with a state-of-the-art cementing technique.

## 4. Antibiotic Elution from ALBCs

Antibiotic elution from ALBCs is a diffusion process. Antibiotic proportion in the cement, surface size, and porosity of the cement are regarded as the most important drivers of antibiotic release from PMMA [11,28]. Table 1 delineates the most relevant factors for antibiotic release that can be directly influenced by the surgeons when using manually loaded and premixed bone cements for spacers.

### 4.1. Choice of Cement

Bone cements differ in their compositions depending not only on the intended use but also on the manufacturer. Commercially available ALBCs have individual proportions of the incorporated antibiotic, are available with antibiotic combinations, and have specific antibiotic elution properties. Therefore, each cement brand and type has its own release behavior. The comparison of medium viscosity Palacos^®^ R+G and high viscosity Palacos^®^ R+G exemplifies the cement specific release: although both cements contain the same proportion of antibiotic, more gentamicin is released from Palacos^®^ R+G.

### 4.2. Geometry of the Cement Body

The geometry of the implanted cement body depends predominantly on the anatomic presuppositions. Duey et al. investigated the release of vancomycin and tobramycin from Simplex^®^ P bone cement by comparing different geometries and volumes. While the antibiotic release did not correlate with specimen volume, a positive linear correlation with the surface area was observed [29]. The outstanding influence of the surface area on antibiotic release is confirmed by the results of Masri et al. Their in vitro study on Simplex^®^ P bone cement showed significant increase of tobramycin elution when the surface area was enlarged, but the cement volume remained constant. In contrast, when the cement volume was reduced but the surface area remained the same, no significant change could be measured [30]. This is because antibiotic elution happens predominantly from the outer layers of the cement. Schurman et al. reported that almost the entire antibiotic was eluted from the superficial 100 micrometer layer while only 19% of incorporated antibiotic was released from the deeper 700 micrometer layer [31]. Thus, although mechanical and surgical considerations have to be taken into account, spacers should be formed with the largest possible surface.

### 4.3. Porosity of the Cured Cement

Pores in the cement matrix increase the surface area and therefore antibiotic elution. As discussed before, porosity of the cement can be modified by adjusting the extent of homogenization of the antibiotic and the powder. Miller et al. produced a highly porous ALBC through the addition of vancomycin chunks. Significantly higher antibiotic release was observed in comparison to a cement for which the antibiotic was thoroughly ground prior to adding it to the cement [32]. Conversely, inhomogeneous antibiotic distribution in the cement matrix is a point of concern because it can lead to inconsistent antibiotic elution [33]. McLaren et al. compared different hand-mixing techniques for manual loading with the corresponding premixed cement formulation. They could not confirm that hand mixing produced a consistently “dissimilar homogeneity of antibiotic distribution” [34]. Lewis et al. compared manually loaded and premixed Cemex (Tecres, Sommacampagna, Italy). While this study reported similar morphologies of the cured cements, the elution rates of the manually loaded cement were 36% lower on average [35]. Because of these in part controversial results, some authors advise against the use of manually mixed ALBCs in general and argue that industrial antibiotic loading provides uniform release kinetics [36].

Irrespective of the loading technique, porosity can be modified by the use of a vacuum mixing system, which is designed to reduce air entrapment in the cement matrix [11].

However, the influence of a vacuum mixing system on antibiotic elution depends on further factors, such as water solubility of the antibiotic, the diffusion gradient, and above all the choice of cement [36]. Meyer et al. compared the effect of a vacuum mixing system on different commercially available gentamicin loaded bone cements. While the antibiotic elution was increased by the use of a vacuum mixer for the ALBCs Palacos^®^ R+G and Cobalt^®^ G-HV (Biomet, Warsaw, IN, USA), it was reduced for Cemex^®^ Genta (Exactech, Gainesville, FL, USA), SmartSet^®^ GMV (DePuy, Warsaw, IN, USA) and VersaBond^®^ AB (Smith & Nephew, London, UK) [37].

Porosity can further be modified in vitro by special additives [38]. Shi et al. reported that added gelatin induced pore formation in PMMA cement [39]. Chen et al. investigated the correlation of PMMA porosity with the particle size and mass fraction of the gelatin [40]. Particularly, CaP is of special interest due to its close connection to bone ongrowth to implants. CaP can increase porosity of bone cements, thereby increasing antibiotic release [41]. However, to date, these porogens are not in routine clinical use. In contrast, calcium carbonate, a biodegradable and soluble substance, is in clinical use. Calcium carbonate is a component of the commercially available bone cement Copal^®^ spacem, which is particularly intended for the use as a spacer. Bitch et al. reported a microporous structure of the hardened cement in contrast to the solid structure of Palacos^®^ R+G. Accordingly, a better elution of several antibiotics was observed for Copal^®^ spacem [42]. However, our own study could not confirm superior elution characteristics of Copal^®^ spacem when a combination of vancomycin and gentamicin was loaded to these two cements [3]. To summarize, the easiest way to tune porosity in the operation theater is by adjusting the homogenization of the antibiotic and the cement.

### 4.4. Antibiotic and Antibiotic Combinations

With manual loading, the choice of antibiotics is restricted to those available as a sterile, heat-stable, and powdery substance with sufficient elution kinetic. Papers on the suitability of antibiotics for manual loading are published regularly. One of the largest overviews is given in Table A1 in the Appendix A [6,14,16,43].

Typically, antibiotics are either eluted with a continuous or with a burst pattern. Gentamicin is a typical example for an antibiotic with a continuous release kinetic [7]. In contrast, vancomycin is typically released in a burst with a high initial release followed by a steep decline. However, the transition from one release kinetic to the other is fluent. Glavez-Lopez et al. compared the elution kinetics of 11 different antibiotics and demonstrated that each antibiotic depicts its own individual release behavior. For example, moxifloxacin showed a longer burst release than vancomycin. While gentamicin is continuously released with almost no decline, meropenem shows a continuously declining release over a long period of time [21].

The combination of antibiotics further influences the elution. Synergistic and antagonistic effects are described. Especially for the combination of gentamicin and vancomycin, synergistic effects are described. Hsie et al. investigated antibiotic elution of gentamicin and vancomycin from Simplex^®^ bone cement. The combination of both antibiotics increased the release of vancomycin by 145%, and of gentamicin by 45%, respectively [20]. Paz et al. investigated the interaction when combining more then two antibiotics. The addition of cefazolin significantly increased vancomycin elution from a gentamicin and vancomycin containing ALBC [4].

However, release kinetics are not only modified by the combination, but also by the relative masses of the combined antibiotics in the cement. A classic example is the significant increase of gentamicin release when the proportion of vancomycin in the cement is raised [11]. Kaplan et al. investigated the combination of daptomycin with tobramycin and observed increased daptomycin elution when the initial amount of tobramycin in the cement was raised [44]. It must be pointed out that the interactions of antibiotic release are subjected to manifold factors, such as the combinations, the proportion in the cement, and the cement brand. Synergistic and antagonistic effects on antibiotic elution can be induced by combining antibiotics [11]. Thus, in vitro testing has to confirm the suitability of the chosen antibiotic combination with the choice of cement before in vivo application.

For clinical use, the simplest way to increase antibiotic elution is to increase its proportion in the cement. Our own study investigated antibiotic elution from vancomycin and gentamicin loaded Palacos^®^ R+G and Copal^®^ spacem. For both cements vancomycin elution was significantly increased when the vancomycin proportion was increased in the cement powder [3].

As discussed above, the proportion of antibiotic in the cement is limited by the reduction of its mechanical properties. Again, it can be summarized that, for preparation of a temporary spacer, manual antibiotic loading up to a cumulative antibiotic proportion of 5–10% in the cement powder is considered advisable [6,16,22,24].

## 5. Future Directions

Currently, PMMA is being further investigated, particularly for its function as a drug carrier. PLGA (Poly-Lactic-co-Glycol-Acid) is an interesting approach to achieving a controlled antibiotic release pattern. Antibiotics can be deposited in microspheres made from PLGA. When these microspheres are added to PMMA or to ALBC, their biodegradability can be modified to achieve a desired release period in vitro ranging from weeks to months [45]. However, it must be kept in mind that mechanical strength remains the most important quality of a PMMA for implant fixation. Adding particles of any kind to a PMMA results in a reduction of its mechanical strength [46]. Future investigations have to determine whether the benefit of antibiotic prophylaxis justifies the risk of resistance formation and aseptic loosening. In contrast, when used as a spacer, the cement’s mechanical strength is less important. So far, the limited in vivo results on pharmacokinetics indicate sufficient antibiotic release of the techniques described above [16]. Therefore, future investigations have to elaborate on the ideal local antibiotic concentration in the context of pathogenic bacterial species, possibilities of surgical debridement, and length of antibiotic treatment.

## 6. Summary

ALBCs are used for prophylaxis and the treatment of periprosthetic infections. For prophylaxis the major concern is a loss of mechanical strength after implantation. Therefore, manually loading cannot be recommended. Instead commercially available ALBCs with consistent material properties should be used with a state-of-the-art cementing technique. For use as a spacer, ALBCs should be tailored by manually loading the spacer with up to 5–10% of a combination of antibiotics targeted to the pathogen. Only examined, crystalline antibiotics should be added to the cement powder after grinding and before initiation of the polymerization process under atmospheric pressure. The spacer should be implanted with the largest possible surface allowed by mechanical and surgical considerations. To date, special bone cements intended for use as a spacer are available. However, the best clinical practice remains to be proven.

## Figures and Tables

**Table 1:** Factors of antibiotic elution for particular consideration during clinical application and their issues.

Factors for Particular Consideration	Issue
(1) Choice of cement	Cement brand and type
(2) Geometry of the cement body	Volume and surface area of the cement
(3) Porosity of the cured cement	Use of a vacuum system, homogenization of antibiotic to the cement powder, porogens in the cement
(4) Antibiotic and antibiotic combinations	Choice of antibiotic, proportion in the cement, combinations

## Appendix A

**Table A1:** Available antibiotics that can be used as spacers; adapted from ICM 2018 [14].

Antibiotic Group	Type of Antibiotic	Activity Against	Dose per 40 g Cement (in Grams)
Aminoglycoside	Tobramycin	Gram-negative bacteria such as Pseudomonas	1 to 4.8
Aminoglycoside	Gentamicin	Gram-negative bacteria—Eschericcia coli, Klebsiella and particularly Pseudomonas aeroginosa. Additionally, aerobic bacteria (not obligate/facultative anaerobes)	0.25 to 4.8
Cephalosporin, 1st gen	Cefazolin	Gram-positive infections, limited Gram negative coverage	1 to 2
Cephalosporin, 2nd gen	Cefuroxime	Reduced gram-positive coverage, improved gram-negative coverage	1.5 to 2
Cephalosporin, 3rd gen	Ceflazidime	Gram-negative bacteria, particularly Pseudomonas	2
Cephalosporin, 4th gen	Cefotaxime	Gram-negative bacteria, no activity against Pseudomonas	2
Cephalosporin, 5th gen	Ceftaroline	Gram-negative bacteria, no activity against Pseudomonas	2 to 4
Fluoroquinolone	Ciprofloxacin	Gram-negative organisms including activity against Enterobacteriaciae	0.2 to 3
Glycopeptide	Vancomycin	Gram-positive bacteria, including methicillin-resistant organisms	0.5 to 4
Lincosamide	Clindamycin	Gram-positive cocci, anaerobes	1 to 2
Macrolide	Erythromycin	Aerobic gram-positive cocci and bacilli	0.5 to 1
Polymyxin	Colistin	Gram-negative	0.24
ß-lactam	Piperacillin—not available Piptzobactam	Gram-negative bacteria (particularly Pseudomonas), Enterobacteria and anaerobes	4 to 8
ß-lactam	Aztreonam	Only gram-negative bacteria	4
ß-lactamase inhibitor	Tazobactam	Gram-negative bacteria (particularly Pseudomonas), Enterobacteria and anaerobes in combination with Piperacillin	0.5
Oxazolidinones	Linezolid	Multidrug-resistant gram-positive cocci such as MRSA	1.2
Carbapenem	Meropenem	Gram-positive and gram-negative bacteria, anaerobes, Pseudomonas	0.5 to 4
Lipopeptide	Daptomycin	Only gram-positive organisms	2
Antifungals	Amphotericin	Most Fungi	200
Antifungal	Voricanazole	Most Fungi	300–600 mg

**Table A2:** Commercially available double loaded bone cements; MRSA: methicillin-resistant Staphylococcus aureus; MRSE: methicillin-resistant Staphylococcus epidermidis adapted from Kuhn et al. 2017 [6].

Antibiotic Groups	Antibiotics	Activity against	Cement Example
Aminoglycoside and Lincosamide	Gentamicin and Clindamycin	Many gram-positive and—negative bacteria (Staphylococcus species, Enterococcus species, Pseudomonas, Escherichia coli, Klebsiella, Proteus	Copal^®^ G+C
Aminoglycoside and Glycopeptide	Gentamicin and Vancomycin	Many gram-positive bacteria (Staphylococcus species, MRSA, MRSE, Enterococcus species	Copal^®^ G+V
Macrolide and Polymyxin	Erythromycin and Colistin	Gram-positive bacteria (Streptococcus species, Staphylococcus species), anaerobic bacteria (Corynebacteria)	Simplex^®^ with Erythromycin/Colistin
